# Red fluorescence protein (DsRed2) promotes the screening efficiency in peanut genetic transformation

**DOI:** 10.3389/fpls.2023.1123644

**Published:** 2023-03-01

**Authors:** Dongxin Huai, Jie Wu, Xiaomeng Xue, Meiling Hu, Chenyang Zhi, Manish K. Pandey, Nian Liu, Li Huang, Dongmei Bai, Liying Yan, Yuning Chen, Xin Wang, Yanping Kang, Zhihui Wang, Huifang Jiang, Yong Lei, Rajeev K. Varshney, Boshou Liao

**Affiliations:** ^1^ Key Laboratory of Biology and Genetic Improvement of Oil Crops, Ministry of Agriculture and Rural Affairs, Oil Crops Research Institute of Chinese Academy of Agricultural Sciences, Wuhan, China; ^2^ Center of Excellence in Genomics and Systems Biology (CEGSB), International Crops Research Institute of the Semi-Arid Tropics (ICRISAT), Hyderabad, India; ^3^ Institute of Industrial Crops, Shanxi Agricultural University, Taiyuan, China; ^4^ State Agricultural Biotechnology Centre, Crop Research Innovation Centre, Food Futures Institute, Murdoch University, Murdoch, Western Australia, Australia

**Keywords:** peanut, DsRed2, genetic transformation, agrobacterium, screening efficiency

## Abstract

Peanut (*Arachis hypogaea* L.), one of the leading oilseed crops worldwide, is an important source of vegetable oil, protein, minerals and vitamins. Peanut is widely cultivated in Asia, Africa and America, and China is the largest producer and consumer of peanut. Genetic engineering has shown great potential to alter the DNA makeup of an organism which is largely hindered by the low transformation and screening efficiency including in peanut. DsRed2 is a reporter gene widely utilized in genetic transformation to facilitate the screening of transformants, but never used in peanut genetic transformation. In this study, we have demonstrated the potential of the red fluorescence protein DsRed2 as a visual reporter to improve screening efficiency in peanut. DsRed2 was firstly expressed in protoplasts isolated from peanut cultivar Zhonhua 12 by PEG, and red fluorescence was successfully detected. Then, DsRed2 was expressed in peanut plants Zhonghua 12 driven by 35S promoter *via Agrobacterium tumefaciens*-mediated transformation. Red fluorescence was visually observed in calli and regenerated shoots, as well as in roots, leaves, flowers, fresh pod shells and mature seeds, suggesting that transgenic screening could be initiated at the early stage of transformation, and continued to the progeny. Upon screening with DsRed2, the positive plant rate was increased from 56.9% to 100%. The transgenic line was then used as the male parent to be crossed with Zhonghua 24, and the hybrid seeds showed red fluorescence as well, indicating that DsRed2 could be applied to hybrid plant identification very efficiently. DsRed2 was also expressed in hairy roots of Huayu 23 *via Agrobacterium rhizogenes*-mediated transformation, and the transgenic roots were easily selected by red fluorescence. In summary, the DsRed2 is an ideal reporter to achieve maximum screening efficiency and accuracy in peanut genetic transformation.

## Introduction

Peanut (*Arachis hypogaea* L.) is an important legume crop of the Fabaceae family, and is widely cultivated in tropical and subtropical regions (Sharma and Bhatnagar-Mathur, 2006). Peanut seed contains various important components with superior nutritional value, such as fat, protein, folate, tocopherol, phytosterols and polyphenolics (Jonnala et al., 2006; De Camargo and Canniatti-Brazaca, 2014). Apart from serving as an oil crop, there is a demand of peanut confectionary preparations such as desserts and snacks (Wang, 2018; Pandey et al., 2020). Therefore, various cultivars are required for the broad purpose of peanut, which can be hardly achieved through the narrow genetic base and conventional breeding methods (Liao, 2017). With the faster developments in the area of biotechnology, genetic engineering by plant transformation has shown great advantages in developing superior cultivars (Krishna et al., 2015). Hence, it is necessary to develop efficient and stable transformation systems of peanut as a foundation for its genetic engineering.

Several methods have been developed to generate highly stable transformed peanut plants, such as particle bombardment, *A. tumefaciens*-mediated transformation and pollen tube pathway system, and the former two methods have achieved relatively greater successes (Eapen and George, 1994; Deng et al., 2001; Zhou et al., 2023). For the particle bombardment method, genes are transferred to embryogenic callus in peanut, and transformed plants are regenerated from somatic embryogenesis (Krishna et al., 2015). For *A. tumefaciens*-mediated transformation, leaflet (Dolce et al., 2018), de-embryonated cotyledon (Hoa et al., 2021; Marka and Nanna, 2021), cotyledonary node (Lamboro et al., 2022) and hypocotyl (Yan et al., 2015) are the main explants, and transgenic plants are regenerated from somatic embryogenesis or organogenesis (Kundu and Gantait, 2018). However, the efficiency of transformation in peanut is still as low as 0.2%–3.3% (Rohini and Rao, 2000), which is determined by the regeneration ability of the explant, host genotype, vectors, screening efficiency and some other factors (Krishna et al., 2015). The low screening efficiency is mostly caused by insufficient selection pressure and improper identification method (Kumar et al., 2011). As a matter of fact, the selection pressure cannot be too strong to screen transformants due to the low regenerability of peanut. Since a few non-transgenic plants may escape and survive, all the regenerated plants need to be further identified (Tiwari and Tuli, 2012). Recently, the most commonly used test method is detection of the exogenous genes by PCR (Marka and Nanna, 2021). However, the accuracy is easily interfered by chimerism, *Agrobacterium*-contamination of the regenerant and aerosol. Therefore, it is significant to find an effective method to accurately distinguish transgenic and non-transgenic plants so as to reduce the workload.

Fluorescent proteins (FPs) are reporter genes widely utilized in genetic transformation of many plant species (Stewart, 2006). Due to their fluorescence characteristics, FPs can be transformed with target genes simultaneously to screen the positive transgenic events. The green fluorescent protein (GFP) isolated from jellyfish (*Aequorea Victoria*) is a frequently used reporter gene in the genetic transformation in monocot and dicot plants (Chung et al., 2000; Yang et al., 2005; Cui et al., 2020). However, its application is largely limited due to the overlapping of its spectral properties with those of several plant pigments (Yang et al., 2005). The red fluorescence protein DsRed2 is a modified form of DsRed from coral (*Discosoma* sp.), which can be easily distinguished from plant cell autofluorescence (Baird et al., 2000). DsRed2 has been successfully used as a visual reporter in numerous plant transformation studies, such as rice, soybean and cotton (Nishizawa et al., 2006; Sun et al., 2018; Toda et al., 2019). These studies have demonstrated that the utilization of FPs can contribute to rapid and accurate screening of stable transformants, which will significantly improve the screening efficiency.

In this study, DsRed2 was used as a visual reporter in peanut genetic transformation, which was delivered into peanut protoplasts by PEG and peanut plants by *A. tumefaciens*-mediated and *A. rhizogene*s-mediated transformation, respectively. The red fluorescence was monitored throughout the whole life of transgenic peanut plants. The screening efficiency with DsRed2 was also investigated. The performance of DsRed2 in transgenic peanut plants was evaluated to assess its ability to serve as a selective marker for peanut biotechnology.

## Materials and methods

### Plant materials

Three peanut cultivars Zhonghua 12 Zhonghua 24 and Huayu 23 were used in this study. Zhonghua 12 (var. *vulgaris*) and Zhonghua 24 (var. *hypogaea*) are developed by Oil Crops Research Institute of the Chinese Academy of Agricultural Sciences, Wuhan, China in 2006 and 2015, respectively. Huayu 23 (var. *hypogaea*) is developed by Shandong Peanut Research Institute, Shandong Academy of Agricultural Sciences, Qingdao, China in 2004.

### Vector construction

A cassette comprising a Nos promoter and 3’ UTR flanking Basta selection marker gene was amplified by PCR from pBinGlyBar1 vector using the following primers with added *Asc*I restriction sites: 5’-CATGGGCGCGCCGCACGCTGCCGCAAGCAC-3’ and 5’-CATGGGCGCGCCCGCGCCGATCTAGTAACA-3’ (the added restriction sites are underlined). Then the *Asc*I digested fragment was inserted into the binary vector pBinGlyRed2 (Huai et al., 2020) containing a 35S promoter-driven DsRed2 gene to generate pBinBarRed (**Figure S1**).

### Protoplast isolation and transfection

Protoplasts were isolated from leaves of 14 days old aseptic seedlings of peanut cultivar Zhonghua 12, which were obtained from seeds sterilized with 0.1% (w/v) HgCl_2_ and cultured in dark. The leaves were cut into 0.5 mm strips, and then dispersed into 30 mL of enzyme solution (2% cellulose R-10, 1% macerozyme R-10, 0.2% pectolase Y-23, 0.1% BSA, 0.6 M mannitol, 10 mM MES (pH 5.7) and 15 mM CaCl_2_) with gently shaking at 40 rpm for 12 hours. After digestion, cells were filtered with a 70 μm nylon meshes, and then washed twice by 30 mL W5 solution (154 mM NaCl, 125 mM CaCl_2_, 5 mM KCl and 2 mM MES, pH5.8). Protoplasts were resuspended in 2 mL W5 solution and stored on ice for 30 mins. Lastly, protoplasts were centrifuged at 100 g for 2 mins, and resuspended in 2 mL MMG solution (0.6 M mannitol, 15 mM CaCl_2_ and 4 mM MES). Cell concentration was measured using a hemocytometer and a light microscope.

Totally 2x10^5^ isolated single protoplasts were suspended in 200 μL MMG solution, and stored on ice for 30 mins. Approximately 20 μg plasmid DNA and 220 μL PEG solution (40% PEG4000, 0.6 M mannitol and 100 mM CaCl_2_) was added, mixed gently and stored at room temperature for 20 mins. After incubation, 1 mL W5 solution was added and stored on ice for 5 mins. Then cells were centrifuged at 100 g for 2 mins, and washed twice by 300 μL W5 solution. The protoplast pellets were resuspended with 1.5 mL W5 solution, and incubated in the dark at room temperature for 48-72 hours. The protoplast pellets were harvested for further analysis.

### *A. tumefaciens*-mediated transformation

The vector pBinBarRed was introduced into *Agrobacterium tumefaciens* strain GV3101 by electro-transformation. A single transformed colony was inoculated into a flask (50 ml) containing sterile 10 ml LB liquid medium supplemented with 50 mg/L kanamycin. The flask was incubated at 28°C for 24 h in a shaker incubator set at 180 rpm until the OD_600_ reached 0.6-0.8. Bacterial suspension was pelleted by centrifugation for 10 min at 250 g and resuspended the cells in the *Agrobacterium* infection medium (2.2g/L MS-B5 and 20 g/L sucrose, OD_600 =_ 0.6-0.8).

**Figure 1:**
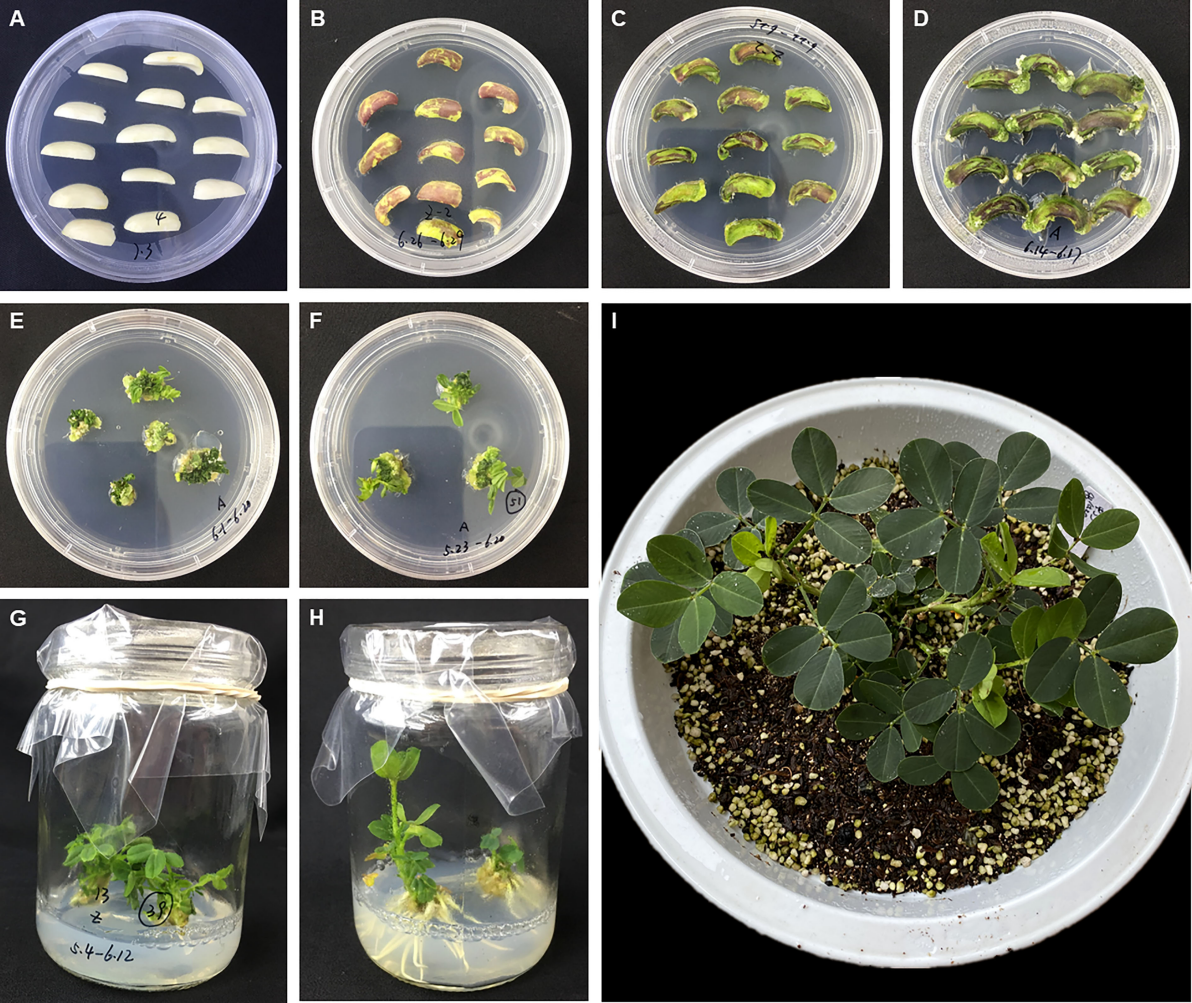
*Agrobacterium tumefaciens*-mediated peanut transformation. **(A)** Cotyledon explants on the Co-cultivation Medium. **(B–D)** Explants on the Shoot Induction Medium after one week **(B)**, two weeks **(C)** and three weeks **(D)**. (**E, F)**. Induced adventitious shoots on the Shoot Elongation Medium after two weeks **(E)** and four weeks **(F)**. **(G, H)** Induced adventitious roots on the Root Induction Medium after two weeks **(G)** and four weeks **(H)**. **(I)** The plantlet was transplanted into soil and maintained in a growth cabinet.

The *A. tumefaciens*-mediated transformation was performed as described by Sharma and Bhatnagar-Mathur (2006) with some modifications. Mature seeds from the peanut cultivar Zhonghua 12 were surface sterilized by rinsing in 75% ethanol for 1 min followed by treatment with 0.1% (w/v) HgCl_2_ for 4 min and then washed 4-6 times with sterile-distilled water and soaked in sterile water overnight. After removing the seed coat, the embryo was removed and each cotyledon was cut into vertical halves which were used as explants. Explants were immersed in the *Agrobacterium* infection medium for 2-5 min, and then transferred onto the Co-cultivation Medium (4.4 g/L MS-B5, 20 g/L sucrose, 4 mg/L BAP, 1 mg/L 2,4-D and 8 g/L agar, pH=5.8) maintained in dark for 72 hours at 26°C (**Figure 1A**). Then explants were transferred onto the Shoot Induction Medium (4.4 g/L MS-B5, 20 g/L sucrose, 4 mg/L BAP, 1 mg/L 2,4-D, 300 mg/L Timentin, 1 mg/L Basta and 8 g/L agar, pH=5.8) and kept at 26°C under 16 h day/8 h dark for 14 days. The explants were transferred onto a fresh medium every 14 days until the shoots come out (**Figures 1B–D**). The shoots were transferred onto the Shoot Elongation Medium (4.4 g/L MS-B5, 20 g/L sucrose, 2 mg/L BAP, 300 mg/L Timentin and 8 g/L agar, pH=5.8) and kept at 26 °C under 16 h day/8 h dark for 14 days. The shoots were transferred onto a fresh medium every 14 days until the shoots grew to 3-4 cm high (**Figures 1E, F**). Subsequently, the elongated shoots were transferred onto the Root Induction Medium (4.4 g/L MS-B5, 20 g/L sucrose and 8 g/L agar, pH=5.8) until the roots grew to 4-5 cm long (**Figures 1G, H**). The plants were transplanted into autoclaved sand-soil (1:1) mixture in plastic pots and maintained in a growth cabinet at 26 °C under 16 h day/8 h dark until the seeds mature (**Figure 1I**).

### *A. rhizogenes-*mediated transformation

The vector pBinBarRed was introduced into *Agrobacterium rhizogenes* strain K599 by electro-transformation. A single transformed colony was grown in 10 mL LB liquid medium containing kanamycin at 50 mg/L and incubated overnight at 28 °C with shaking at 180 rpm until the OD_600_ reached 0.6–0.8. The bacterial cells were collected by centrifugation for 10 min at 250 g and resuspended in the *A. rhizogenes* solutions (2.2 g/L MS, 20 g/L sucrose and 100 μM AS, OD_600 =_ 0.6-0.8).

**Figure 2:**
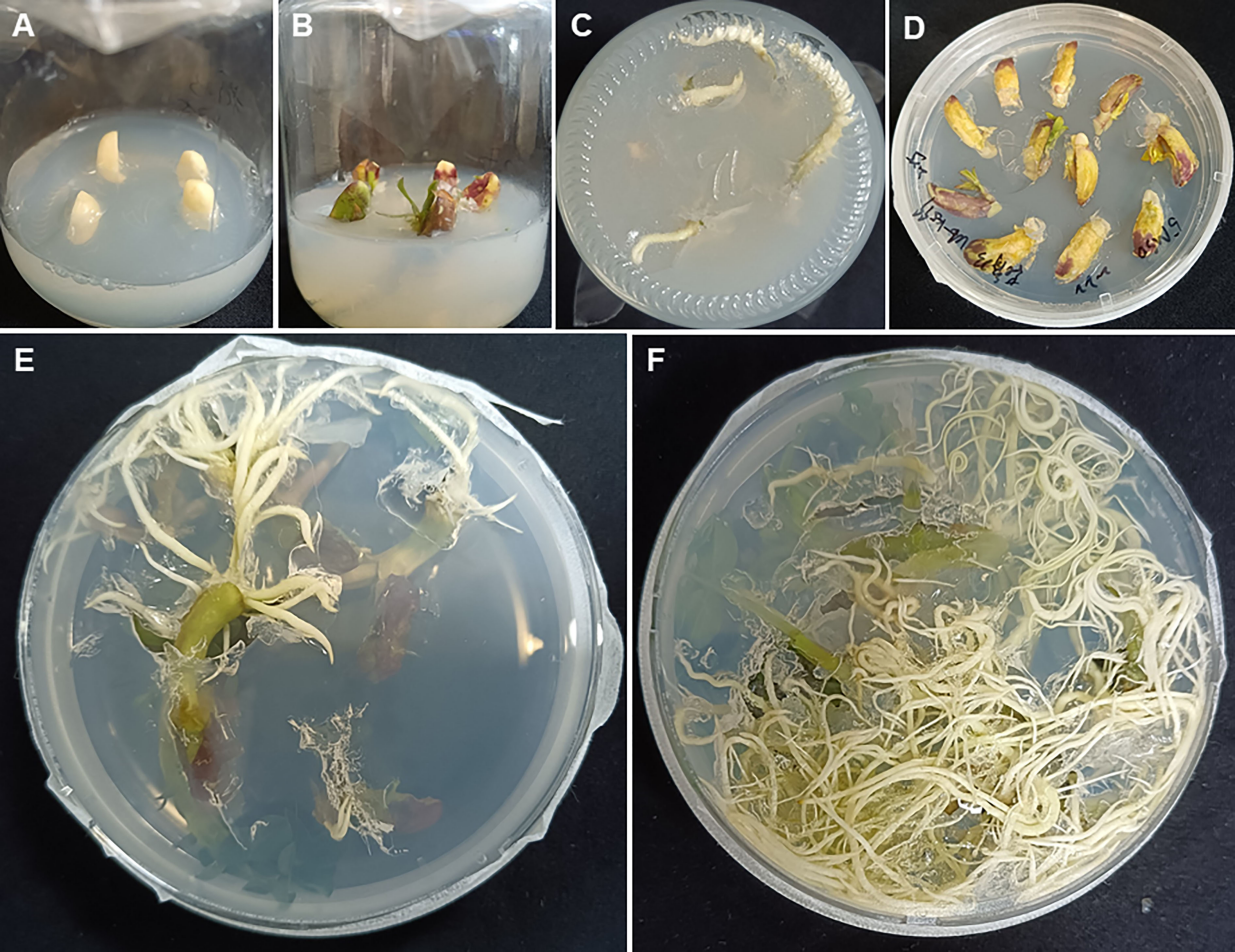
*Agrobacterium rhizogenes*-mediated peanut transformation. **(A)** Surface sterilized seeds on ½MS medium. **(B, C)** One-week old seedling for inoculation. **(D)** Explants on the co-cultivation medium. **(E, F)** Induced hairy roots appearing two weeks **(E)** and four weeks **(F)** after inoculation.

Mature seeds from the peanut cultivar Huayu 23 were surface sterilized and germinated on ½MS medium (**Figures 2A–C**). After 1 week, the radicle and hypocotyl were cut from each seedling and the remaining portion was used as explant. Explants were dipped in the *A. rhizogenes* solutions and incubated for 2-5 min, and then transferred onto the co-cultivation medium (4.4 g/L MS, 20 g/L sucrose, 50 μM AS and 8 g/L agar, pH=5.8) maintained in dark at 26°C for 3 days (**Figure 2D**). Subsequently, explants were transferred onto the hairy root induction medium (4.4 g/L MS, 20 g/L sucrose, 300 mg/L Timentin and 8 g/L agar, pH=5.8) and kept at 26 °C under 16 h day/8 h dark until the root grow well (**Figures 2E, F**).

### Fluorescence observation

The fluorescence in protoplast cells was examined under a laser-scanning confocal microscope (Olympus FV 10-ASW). The fluorescence in callus and samples form transgenic plants were observed using a green-light hand-held lamp (LUYOR, China), with a red camera filter lens.

### DNA extraction and PCR analysis

Total genomic DNA was isolated from young leaves of transgenic plantlets and wild-type peanut plants using a EasyScript Plant Genomic DNA Kit (Transgen, China). To detect the presence of *DsRed2* gene, the following primers were used for PCR amplification: 5’- TTCAAGGTGCGCATGGAG-3’ and 5’-CGTTGTGGGAGGTGATGT-3’. The amplification cycle consisted of denaturation at 94 °C for 1 min, primer annealing at 58 °C for 1 min, and primer extension at 72 °C for 1 min. After 30 repeats of the thermal cycle and final extension 72 °C for 10 min, amplification products were analyzed on 1% agarose gels. The putative PCR product was 577 bp.

### RNA extraction and real-time PCR analysis

Total RNA was extracted from young leaves of transgenic plantlets and wild-type peanut plants using TRIzol reagent (Sigma, USA). Reverse transcription was implemented using SuperScript IV First-Strand Synthesis System (Invitrogen, USA). Real time PCRs were performed on a Bio-Rad CFX96 Real-Time system using SYBR Green as fluorescent dye. The peanut *actin* gene was used as internal control using primers: 5’- TAAGAACAATGTTGCCATACAGA-3’ and 5’-GTTGCCTTGGATTATGAGC-3’. The primers for *DsRed2* gene were: 5’-GTACGGCTCCAAGGTGTACG-3’ and 5’-TAGATGAAGCAGCCGTCCTG-3’.

### Crossing and hybrid identification

A high oleate peanut cultivar Zhonghua 24 was pollinated with the pollens from transgenic Line 1 (T_1_), which were developed from normal oleate cultivar Zhonghua 12. As the difference between the oleic acid contents in parents were caused by mutations in *AhFad2* genes (Chu et al., 2009; Pandey et al., 2014), primers for *AhFad2* genes were used for hybrid identification: 5’- CACTAAGATTGAAGCTC-3’ and 5’-CACTAAGATTGAAGCTC -3’. A 500 bp fragment was amplified by PCR and sequenced by Sanger sequencing.

## Results

### Evaluation of DsRed2 in peanut protoplast transformation

**Figure 3:**
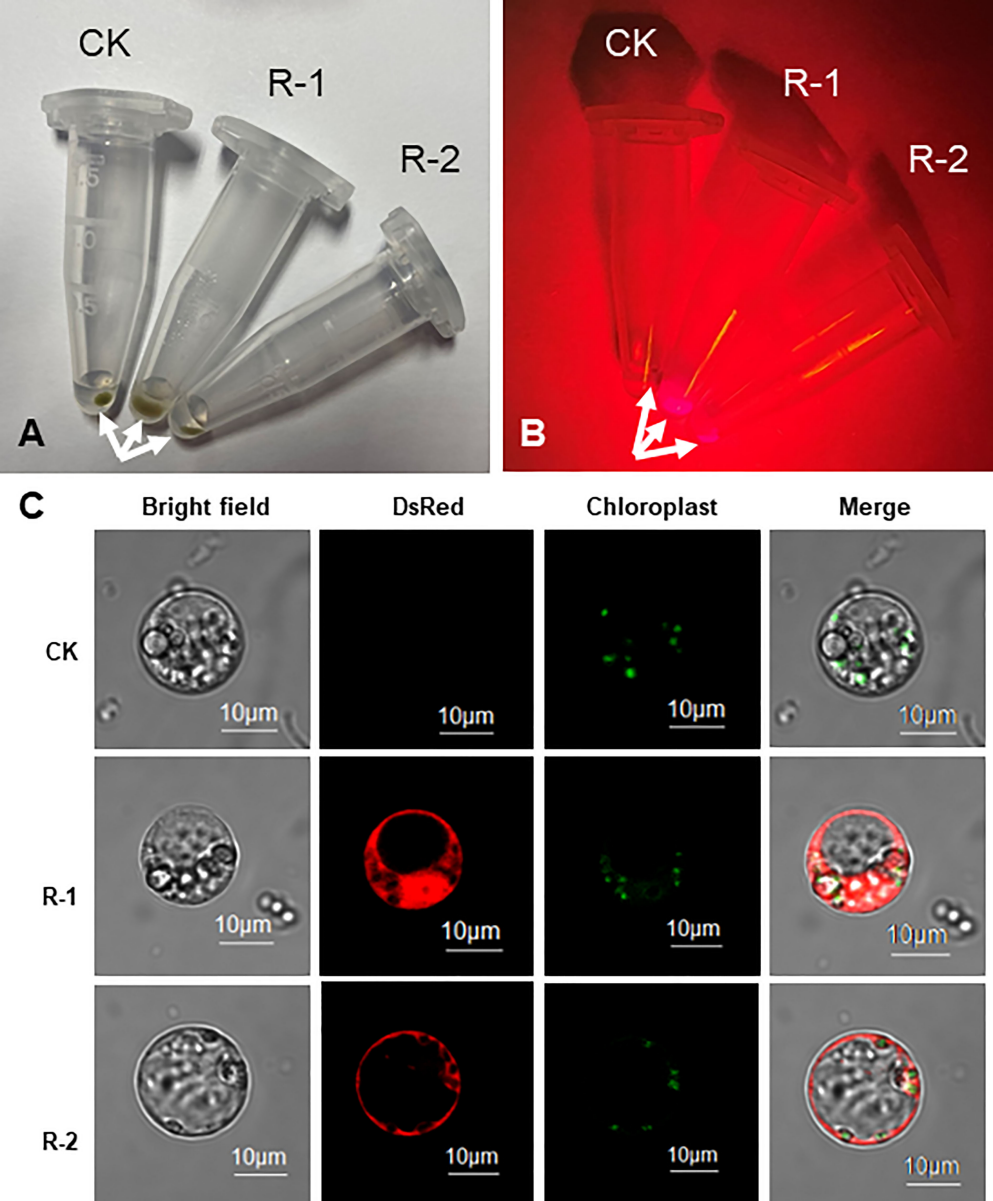
*DsRed2* gene expression in peanut protoplast. **(A, B)** Centrifuged peanut protoplasts at the bottom of Eppendorf tubes under white light **(A)** and green light **(B)**. Arrows indicate the centrifuged protoplasts. **(C)** Characteristic features of organelles in peanut protoplasts. CK, peanut protoplast transformed with pBinGlyBar1; R-1 and R-2, peanut protoplast transformed with pBinBarRed.

Firstly, the *DsRed2* gene was expressed in protoplasts isolated from peanut leaves. After transformation, the protoplasts were centrifuged to the bottom of tubes (**Figure 3A**). Under green light, the transformed protoplasts R-1 and R-2 showed bright red fluorescence compared with CK (**Figure 3B**). Under a laser confocal microscope, red fluorescence was detected in R-1 and R-2, but not in CK (**Figure 3C**). These results indicated that DsRed2 could be used as a reporter in peanut protoplast transformation.

### Evaluation of DsRed2 in *A. tumefaciens*-mediated peanut transformation

**Figure 4:**
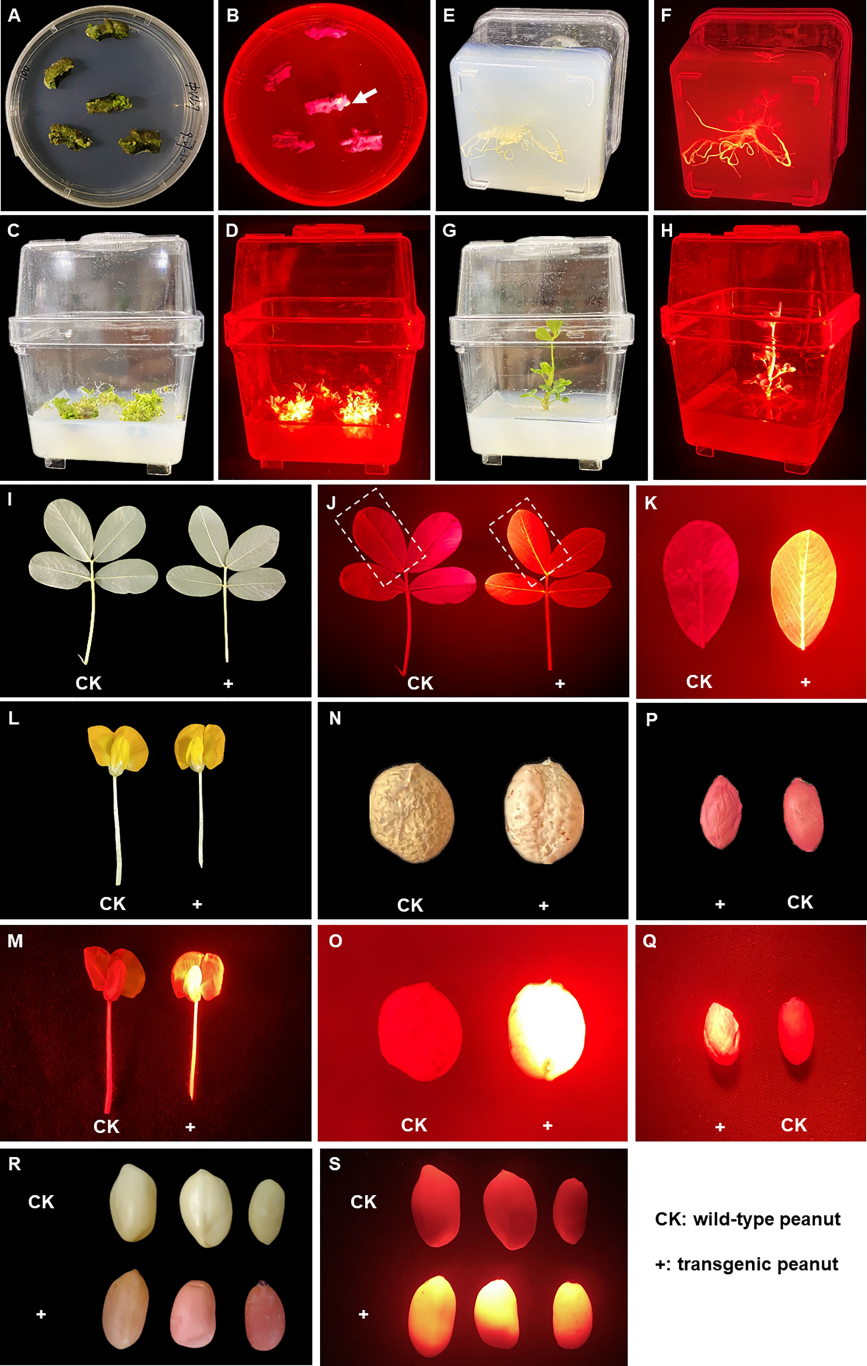
*DsRed2* gene expression in callus, different tissues and organs *via A. tumefaciens*-mediated peanut transformation. **(A, B)** *DsRed2* expression in peanut callus under white light **(A)** and green light **(B)**. The arrow indicates the transformed callus with red fluorescent. **(C, D)** *DsRed2* expression in induced shoots under white light **(C)** and green light **(D)**. **(E, F)** *DsRed2* expression in induced roots under white light **(E)** and green light **(F)**. **(G, H)** *DsRed2* expression in transgenic plantlet under white light **(G)** and green light **(H)**. **(I–K)** *DsRed2* expression in mature leave under white light **(I)** and green light **(J, K)**. **(L, M)** *DsRed2* expression in flower under white light **(L)** and green light **(M)**. **(N, O)** *DsRed2* expression in pod under white light **(N)** and green light **(O)**. **(P, Q)** *DsRed2* expression in seed under white light **(P)** and green light **(Q)**. **(R, S)** *DsRed2* expression in seed without testa under white light **(R)** and green light **(S)**.

The *DsRed2* gene was constitutively expressed in peanut *via A. tumefaciens*-mediated transformation. Under green light, red fluorescence could be observed at very early stage of callus formation (**Figure 4B**), and became increasingly pronounced with callus age. Upon the emergence of shoots, the transgenic shoots were selected by red fluorescence (**Figure 4D** and **Figure S2B**). The shoots showing red fluorescence developed into plantlets with red fluorescence (**Figure 4H**). The whole plant exhibited bright red fluorescence under green light at both vegetative and reproductive stages, including the root (**Figure 4F**), leaf (**Figure 4K**), flower (**Figure 4M**), pod shell (**Figure 4O**) and seed (**Figure 4Q**). Under white light, after removal of the seed coat, the color of embryo and cotyledon was red in transgenic seeds, but was white in CK (**Figure 4R**), allowing very easy discrimination of the transgenic seeds with naked eyes. The T_1_ progeny of DsRed2 peanut exhibited the same morphological characteristics as T_0_ generation, indicating that the fluorescence can be inherited by subsequent generations. Hence, DsRed2 can serve as a stable reporter in *A. tumefaciens*-mediated peanut transformation.

### Molecular analysis of putative transgenic plants

**Figure 5:**
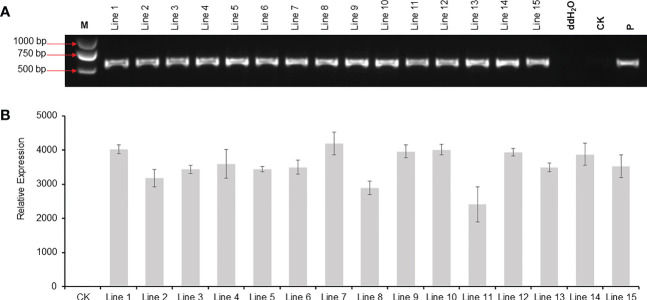
PCR and RT-PCR analysis of *DsRed2* gene in transgenic plants. **(A)** PCR amplification of *DsRed2* in transgenic plantlets. **(B)** The expression level of *DsRed2* in transgenic plantlets. Lines 1-15, transgenic lines with red fluorescent; CK, wild-type plant; P, plasmid of pBinBarRed.

A total of 52 T_0_ transgenic plantlets were obtained, and 15 of which were tested by PCR. As shown in **Figure 5A**, all the 15 lines harbored the DsRed2 gene. Compared with the transgenic lines without the DsRed2 reporter, the positive rate increased from 56.9% (**Figure S3**) to 100% (**Figure 5A**). The expression levels of *DsRed2* were detected in leaves of the 15 transgenic lines by qRT-PCR. As expected, the expression of *DsRed2* was not detectable in the non-transformed control, while was detected in transgenic lines (**Figure 5B**). Hence, the application of DsRed2 in peanut transformation can greatly increase the screening efficiency and improve the positive rate.

### Evaluation of DsRed2 in hybrid identification

**Figure 6:**
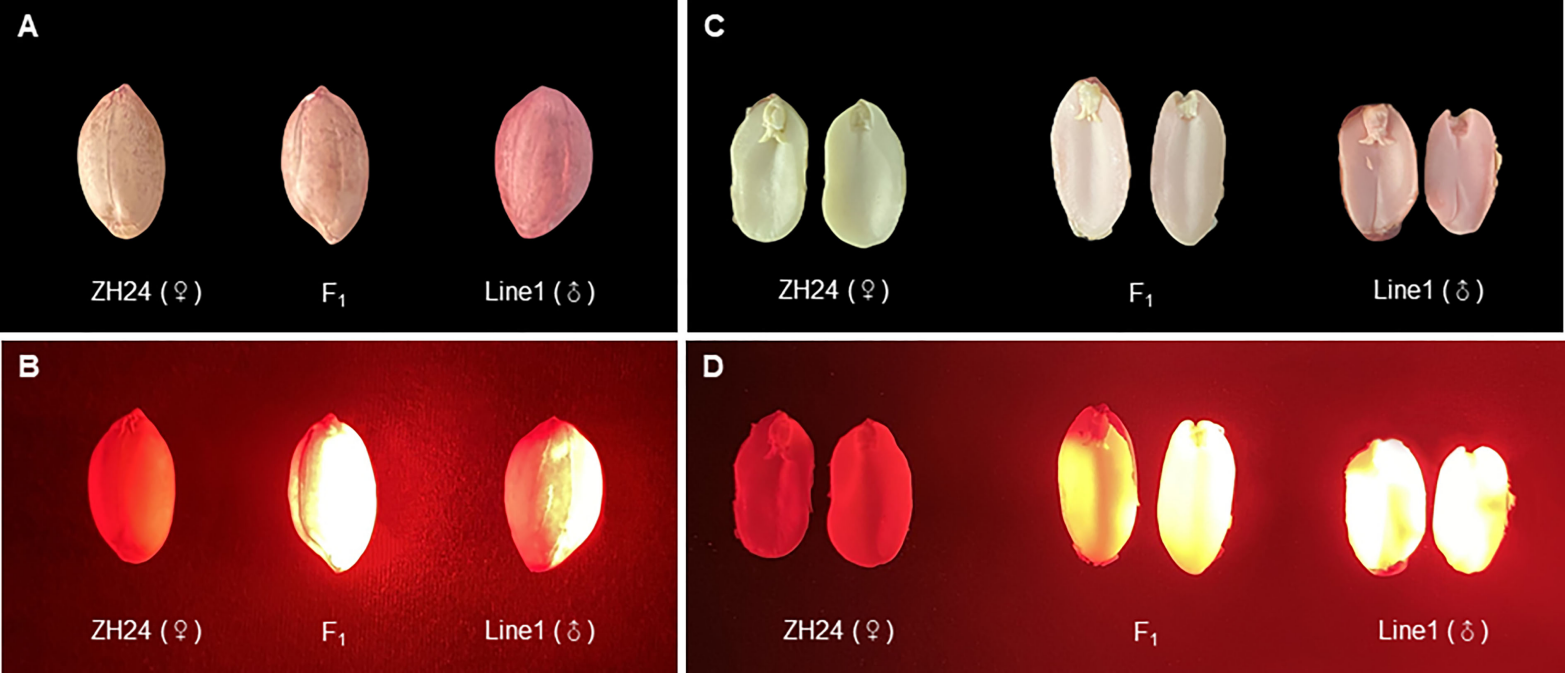
F_1_ hybrids generated from a cross between *DsRed2* transgenic plants and Zhonghua 24 cultivar. **(A, B)** Seeds of F_1_ hybrid, the female parent Zhonghua 24 and the male parent *DsRed2* transgenic Line1 under white light **(A)** and green light **(B)**. **(C, D)** The embryo and cotyledon of F_1_ hybrid, the female parent Zhonghua 24 and the male parent *DsRed2* transgenic Line1 under white light **(C)** and green light **(D)**.

The transgenic Line 1 with DsRed2 was used as the male parent (with normal oleate trait) to be crossed with the high-oleate peanut cultivar Zhonghua 24, and 28 F_1_ hybrid seeds were obtained. The seed testa color was pink in the female parent Zhonghua 24, while red in the male parent transgenic Line 1. The seed testa color of hybrid was pink, suggesting that the F_1_ seeds were obtained from the female parent instead of mixing with seeds from the male parent (**Figure 6A**). The color of embryo and cotyledon was white in the female parent, red in the male parent, while lightly red in the hybrid seeds, indicating that the DsRed2 gene was transferred into hybrid plants (**Figure 6C**). Furthermore, red fluorescence was detected in both hybrid seeds and male parent seeds, but not in female parent seeds under green light (**Figures 6B and D**). Sanger sequencing results revealed that the genotypes of *AhFAD2* genes controlling the oleic content in all the 28 red seeds were heterozygous, indicating that they were true hybrids (**Figure S4**). All these results suggested that DsRed2 could be used as a reliable reporter in peanut hybrid identification.

### Evaluation of DsRed2 in *A. rhizogenes*-mediated peanut transformation

**Figure 7:**
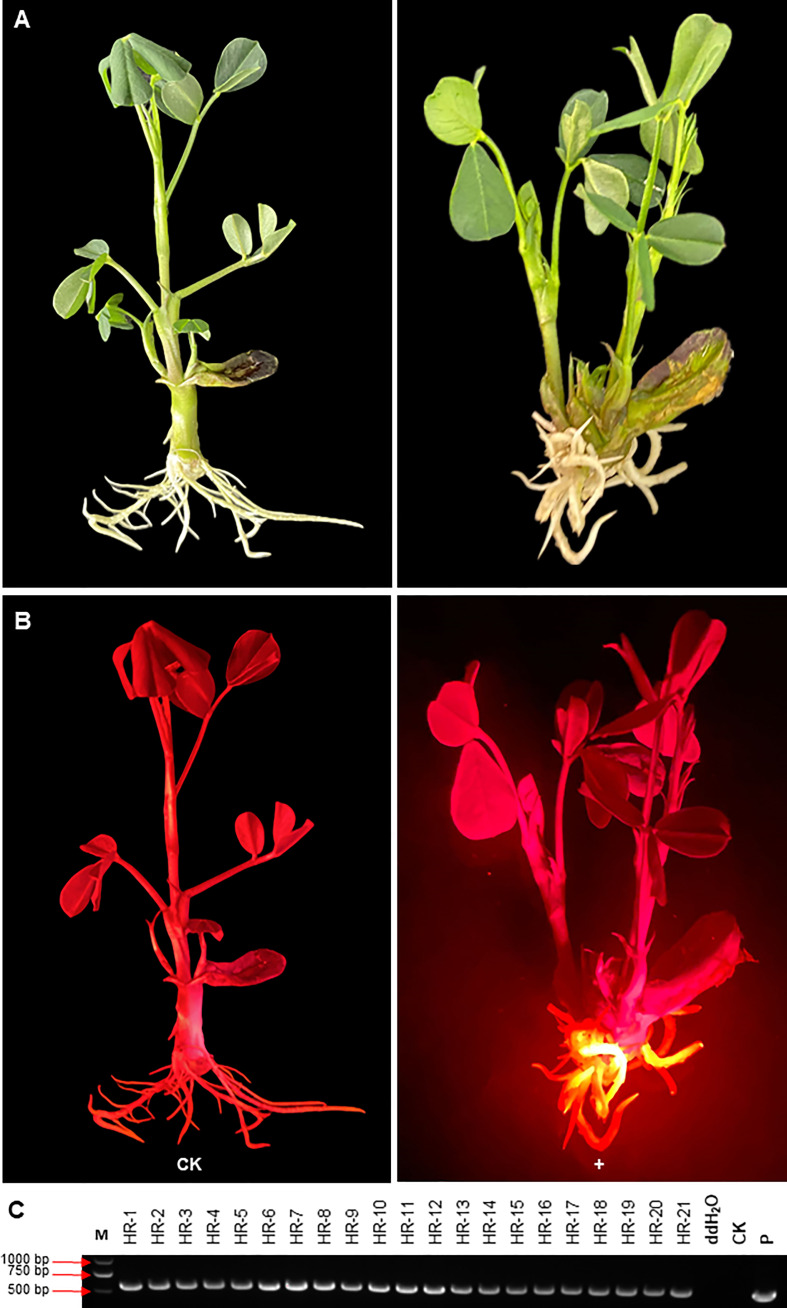
*DsRed2* gene expression in hairy roots *via A. rhizogenes*-mediated peanut transformation.**(A, B)** *DsRed2* expression in hairy roots under white light **(A)** and green light **(B)**. **(C)** PCR amplification of *DsRed2* in transgenic hairy roots. HR-1 to HR-21, transgenic plantlets with red hairy roots; CK, wild-type plant; P, plasmid of pBinBarRed.

The selection effect of DsRed2 in *A. rhizogenes*-mediated peanut transformation was also evaluated (**Figure 7a**). As shown in **Figure 7B**, red fluorescence can only be observed in induced hairy roots but not in other parts under green light. Twenty-one plantlets with transgenes were selected by red fluorescence, and verified by PCR. The positive rate of transgenic hairy root was also reached to 100% (**Figure 7C**). These results also demonstrated that DsRed2 is also an ideal reporter in *A. rhizogenes*-mediated peanut transformation.

## Discussion

A highly efficient transformation system is critical for the improvement of crops including peanut. Although several peanut transformation systems have been established, their transformation efficiency and reproducibility are still largely inadequate. Hence, it is necessary to improve the transformation efficiency by optimizing the transformation parameters such as plant genotype, inoculation, co-culture conditions and selective reporters (Krishna et al., 2015). GFP was used as a reporter in peanut genetic transformation by particle bombardment. Green fluorescence was observed in somatic embryo, root and leave, but green fluorescence in shoot tissues was confounded with fluorescence from chlorophyll (Joshi et al., 2005). DsRed2 is a red fluorescent protein from corals of the *Discosoma* genus, which has been used successfully in research on animals, fungi and plants (Baird et al., 2000). In this study, DsRed2 was used as a visible selective reporter and its performance in peanut genetic transformation was evaluated. DsRed2 was successfully expressed in peanut protoplasts and plants (**Figures 3** and **4**), suggesting that it can be steadily expressed and inherited in peanut. Moreover, a red color could be clearly observed with naked eyes in embryo and cotyledon without the help of any instrument (**Figures 4R** and **6C**), which can greatly facilitate the screening of transgenic seeds in the progeny and hybrids. Hence, DsRed2 can serve as an effective visual reporter gene for genetic transformation of peanut.

Compared with low identification accuracy of transgenic peanut plant is an important factor limiting the screening efficiency in addition to resources wastage (Mallikarjuna and Varshney, 2014). In previous studies, the highest positive rate of peanut transformation was 90% (Krishna et al., 2015). In this study, application of DsRed2 significantly increased the screening efficiency and improved the positive rate from 56.9% to 100% (**Figures 5** and **S3**), which is the highest score in peanut transformation. In previous studies, the most common identification method is detection of the exogenous genes by PCR and should be performed after acquired regenerated plants (Marka and Nanna, 2021). In this study, as red fluorescence could be observed at a very early stage of callus formation (**Figure 4B**), the screening could be started very early as well, reducing much labor and resource consumption. Furthermore, identification with PCR requires a special instrument and reagents, but screening with DsRed2 requires only a lamp and a filter, which is much cheaper and easy to operate. Therefore, screening with DsRed2 in peanut transformation can promote the efficiency and save resources to some extent.

In the present study, the transgenic peanut plants were generated *via* organogenesis in the *A. tumefaciens*-mediated transformation system. Red fluorescence was detected in all tissues of transgenic plants at both vegetative and reproductive stages, including the root (**Figure 4F**), leaf (**Figure 4K**), flower (**Figure 4M**), pod shell (**Figure 4O**) and seed (**Figure 4Q**). Interestingly, no chimera was detected in the transgenic plants. Relative to somatic embryogenesis, organogenesis protocol is easier to handle and more time-saving, but may lead to the generation of chimera in some researches (Tiwari and Tuli, 2012). The red fluorescence screening can effectively avoid the occurrence of chimera. Therefore, application of the DsRed2 reporter in organogenesis protocol can greatly simplify the operation and increase the efficiency of peanut transformation.

Genome-editing technologies have revolutionized plant research and exhibit great potential in the improvement of crops (Kausch et al., 2019). In this study, an efficiently and stable transformation system was successfully established for peanut, which may be widely used in metabolic engineering and genome-editing of peanut and greatly facilitate future peanut improvement.

## Data availability statement

The original contributions presented in the study are included in the article/**Supplementary Material**. Further inquiries can be directed to the corresponding authors.

## Author contributions

DH, YL and BL conceived and designed the experiments. HJ and DB supplied the peanut lines. JW, XX, MH, CZ, NL, LH, LY, YC, XW, YK and ZW performed the experiments. DH, JW, and XX analyzed the data. DH and JW wrote the manuscript. DH, PB, MKP, YL, RKV and BL contributed in data interpretation and revision of the manuscript. All authors contributed to the article and approved the submitted version.

## Supplementary material

Supplementary Figure 1 Construct of pBinBarRed. *DsRed2* was inserted into pBinBarRed vector. Constitutive and seed-specific promoters, 3’ UTR sequences and arrangements of cassettes are also shown.

Supplementary Figure 2 Selection of transgenic shoots using *DsRed2* gene. **(A)** Transgenic shoots and non-transgenic shoots under the white light. **(B)** Transgenic shoots and non-transgenic shoots under the green light.

Supplementary Figure 3 PCR analysis of *Bar* and *Luc* genes in transgenic plants without DsRed2 selection reporter. **(A)** PCR amplification of *Bar* gene in transgenic plantlets. **(B)** PCR amplification of *Luc* gene in transgenic plantlets. +, transgenic line which harboring both of *Bar* and *Luc* genes; -, non-transgenic line which possessing one or none of *Bar* and *Luc* genes. Among 65 putative plants, 37 plants were identified as transgenic plants, so the positive efficiency was 65.9%. 1-65, putative transgenic plants (T_0_); CK, wild-type plant; P, plasmid of pBinGlyBar1.

Supplementary Figure 4 Sequence from a PCR product of *AhFAD2* genes in hybrids (F_1_). The mutant allele of *AhFAD2A* had a 1-bp substitution (G:C→A:T) at position 448 after the start codon; the mutant allele of *AhFAD2B* had a 1-bp insertion (A:T) at position 442 after the start codon. The true hybrid (F_1_) was identified by containing double peaks (G/A) at position 442 bp (*AhFAD2B*) and triple peaks (G/A/C) at position 448 bp (*AhFAD2A*) after the start codon. A, green; T, red; C, blue; G, black.
