# Supplementary figures and images for: Red fluorescence protein (DsRed2) promotes the screening efficiency in peanut genetic transformation
Source: Front Plant Sci. 2023 Mar 1;14:1123644. doi: 10.3389/fpls.2023.1123644 (PMC10014910; doi:10.3389/fpls.2023.1123644)

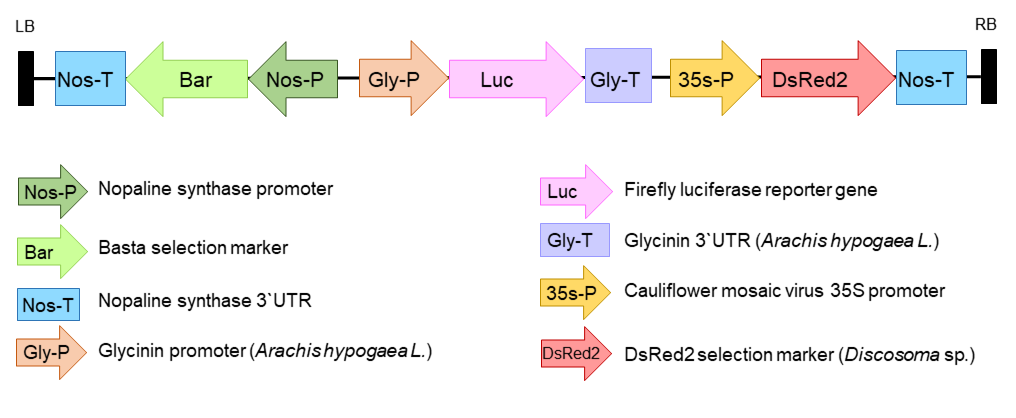

Supplement: Supplementary Figure 1 — Construct of pBinBarRed. DsRed2 was inserted into pBinBarRed vector. Constitutive and seed-specific promoters, 3’ UTR sequences and arrangements of cassettes are also shown. [file Image_1.tif]

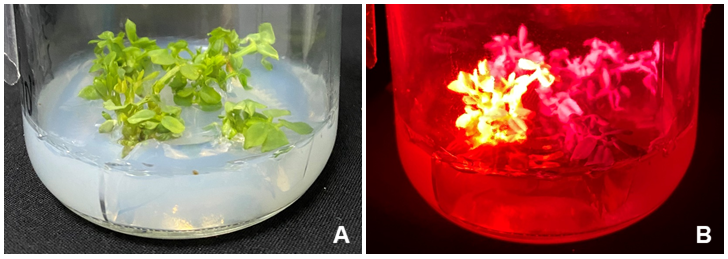

Supplement: Supplementary Figure 2 — Selection of transgenic shoots using DsRed2 gene. (A) Transgenic shoots and non-transgenic shoots under the white light. (B) Transgenic shoots and non-transgenic shoots under the green light. [file Image_2.tif]

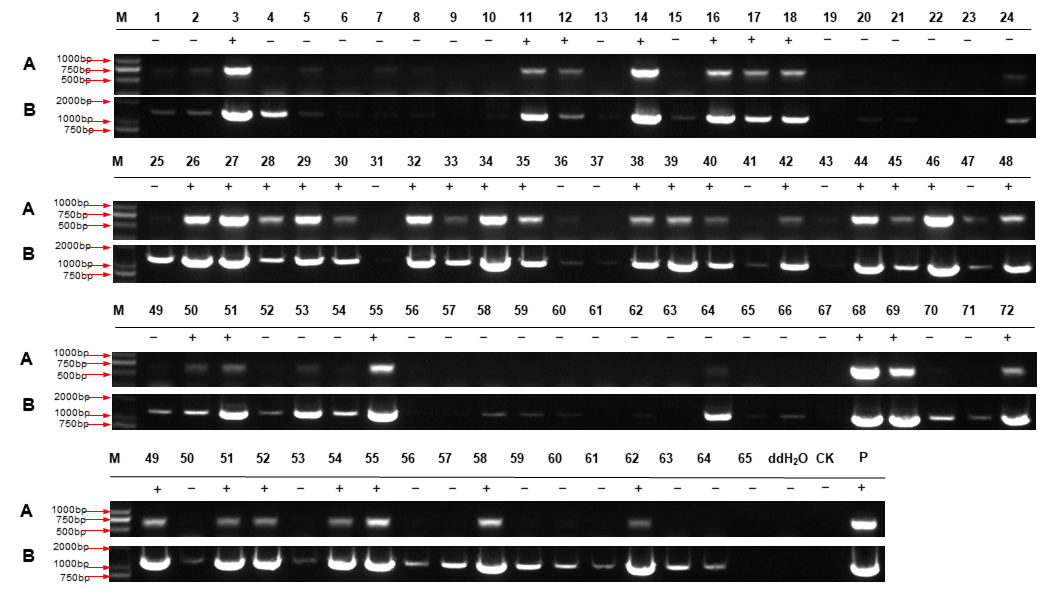

Supplement: Supplementary Figure 3 — PCR analysis of Bar and Luc genes in transgenic plants without DsRed2 selection reporter. (A) PCR amplification of Bar gene in transgenic plantlets. (B) PCR amplification of Luc gene in transgenic plantlets. +, transgenic line which harboring both of Bar and Luc genes; -, non-transgenic line which possessing one or none of Bar and Luc genes. Among 65 putative plants, 37 plants were identified as transgenic plants, so the positive efficiency was 65.9%. 1-65, putative transgenic plants (T0); CK, wild-type plant; P, plasmid of pBinGlyBar1. [file Image_3.tif]

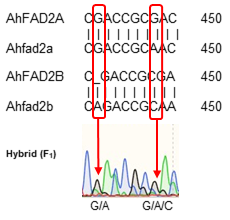

Supplement: Supplementary Figure 4 — Sequence from a PCR product of AhFAD2 genes in hybrids (F1). The mutant allele of AhFAD2A had a 1-bp substitution (G:C→A:T) at position 448 after the start codon; the mutant allele of AhFAD2B had a 1-bp insertion (A:T) at position 442 after the start codon. The true hybrid (F1) was identified by containing double peaks (G/A) at position 442 bp (AhFAD2B) and triple peaks (G/A/C) at position 448 bp (AhFAD2A) after the start codon. A, green; T, red; C, blue; G, black. [file Image_4.tif]
